# Intracranial pulse wave velocity using 4D flow MRI: method comparison and covariate analysis

**DOI:** 10.1098/rsfs.2024.0036

**Published:** 2025-04-04

**Authors:** Sergio Dempsey, Soroush Safaei, Samantha J. Holdsworth, Gonzalo D. Maso Talou

**Affiliations:** ^1^ Auckland Bioengineering Institute, University of Auckland, 70 Symonds Street, Auckland 1010, New Zealand; ^2^ Mātai Medical Research Institute, 466 Childers Road, Gisborne 4010, New Zealand; ^3^ Faculty of Medical and Health Sciences & Centre for Brain Research, University of Auckland, Auckland, New Zealand

**Keywords:** pulse wave velocity, arterial stiffness, magnetic resonance imaging, 4D flow, cerebral blood flow

## Abstract

Intracranial pulse wave velocity (PWV) offers the potential to enhance neurovascular care when evaluating cerebrovascular disease. Using 4D flow MRI, we measured PWV in the intracranial vasculature stemming from the internal carotids and basilar arteries using three popular techniques: cross-correlation, waveform optimization and time-to-upstroke which have all been used intracranially, but never compared. Near-perfect agreement between cross-correlation and waveform optimization methods was observed, while the time-to-upstroke method estimated a significantly larger PWV and was more prone to non-physiological values in a cohort of 21 healthy individuals aged 48 ± 18 years. We then analysed our cohort PWV using an ensemble approach given the current lack of methodological consensus. This analysis identified two consistent findings. First, internal carotids measure significantly higher PWV than basilar vascular networks (3.64 ± 1.47 versus 2.53 ± 1.39 m s^−1^). Second, in our cohort, intracranial PWV was age-independent. We hypothesize that age independence is a healthy physiological trait to minimize microvascular strain, protecting the integrity of the peripheral bed throughout ageing and cardiac pulsatile deformation. The cause for apparent age independence remains unknown. We also identified that previous work on intracranial PWV is likely biased towards the extracranial vasculature, which may explain the study differences in PWV magnitude and the age-dependent nature.

## 1. Introduction

Pulse wave velocity (PWV) measures the speed of the cardiac pressure wave and is hypothesized to reflect arterial stiffness [1]. Therefore, altered PWV has been canonically considered a reliable biomarker for atherosclerosis, vascular ageing and arterial calcification and to characterize the risk of hypertension, end-organ damage and vascular disease [2]. Recent work has also identified the potential importance of elevated PWV as a risk factor for dementia [3,4] and other measures of neurodegeneration [5]. PWV can be approximated globally using common carotid–femoral and brachial–ankle ultrasound techniques [2], but this may not be appropriate to infer cerebrovascular trends where the vasculature is highly modulated [6]. To measure intracranial PWV, magnetic resonance imaging (MRI) is the common option as ultrasound has limitations in the imaging of cerebral vessels due to the high acoustic impedance of the cranium and measuring distance required for proper PWV measurement [7], limiting pathological differentiation, interpretation and precision for brain applications.

Historically, MRI work focused on developing techniques to measure PWV in the aortic arch (see the review of Wentland *et al*. [1]). As scanning resolutions became finer, these methodologies have been applied closer to the brain, but studies are sparse [4,5,8–11]. A two-point time-to-foot ($c_{tf}$) approach using two-dimensional (2D) phase contrast (PC) was used in the internal carotid artery (ICA) by Kröner *et al*. [8], who identified that ICA and aortic arch PWVs were similar. The mean of a multipoint cross-correlation ($c_{cc}$) and $c_{tf}$ technique was used in the carotids by Peper *et al*. [9] with multiple 2D PC slices, which showed good agreement with ultrasound techniques at the same location. A two-point time-to-upstroke ($c_{tu}$) approach was used in the ICAs by Rivera-Rivera *et al*. [4] (one point intracranial, one point extracranial), who identified increased PWV in mild cognitive impairment and Alzheimer’s disease cohorts compared to age-matched controls. It is unclear why a two-point methodology was used as multipoint regression has been shown to be more robust to noise [1] and three-dimensional time resolved PC (4D) flow data were available. Since reviewed by Wentland *et al*. [1], a new technique, we describe as waveform optimization ($c_{wo}$), has been developed to measure cerebral PWV by Björnfot *et al*. [10], which leverages data of an entire vascular tree. We note that $c_{tu}$ and $c_{wo}$ have not been compared with more established methods like $c_{cc}$ and $c_{tf}$.

We also note that no groups have reported vascular differences in PWV between the left and right ICA, nor is there any reported PWV for the basilar arterial (BA) network, which would have been possible in 4D flow studies [4,5,10,11]. Since the distance from the heart to the anterior-temporal and posterior vascular territories in the brain is different, as well as their local anatomy and potentially mechanical vascular properties, we hypothesize that a separate assessment for each territory perfused by the ICA and basilar arteries would lead to a more insightful physiologic description of the brain. The role and value of a differential territory assessment have not been realized clinically, but could identify territory-based vascular risk factors, as discussed in other works [12]. Therefore, we propose that network differences in PWV between left- and right-ICAs or BA-perfused territories should be reported when possible. Björnfot *et al*. [5,10] and Rivera-Rivera *et al*. [11] measure a mixed ICA PWV, Rivera-Rivera *et al*. [4] only reported the average PWV of both ICAs, Peper *et al*. [9] and Kröner *et al*. [8] only report left ICA. Since there is currently no consensus on ideal technique, guided by [1,13,14], we propose to report on vascular territory differences using an ensemble approach between three PWV techniques to avoid outliers when a specific technique fails (non-physiological PWV) and to average the predictions when they agree.

Together, this work sets out to: (i) evaluate methodological differences between $c_{wo}$, $c_{tu}$ and $c_{cc}$ intracranial PWV techniques and (ii) contribute to the sparse analysis of vascular territory (anterior ICA versus posterior BA vascular network) differences in PWV in the brain. To evaluate differences between territories, they are compared against anticipated and potential covariates that could influence PWV, namely age, heart rate (HR), cerebral blood flow (CBF), velocity and vessel size. All is achieved by analysing 4D flow MRI, an emerging sequence for analysing cerebrovascular haemodynamics [15].

## 2. Material and methods

### 2.1. Subjects and scanning protocol

We retrospectively analysed 21 healthy participants (aged 46 ± 18 years, span 22–75 years; 10 female, 11 male). The inclusion criteria were those without a history of brain trauma, hypertension, cognitive impairment or cardiovascular disease. Ethical approval was obtained from the New Zealand Health and Disability Ethics Committee (HDEC9430) and the University of Auckland Ethics Committee (AHREC1006). The study was carried out in accordance with the guidelines of the 1964 Declaration of Helsinki and its subsequent amendments. All participants provided written informed consent.

Scanning took place on a 3 T MRI scanner (GE SIGNA Premier; General Electric Healthcare, Milwaukee, WI, USA) using an AIR™ 48-channel head coil with a *k*-adaptive-*t* autocalibrating reconstruction for cartesian sampling (kat-ARC) 4D flow protocol [16]. The resolution was acquired at 1 mm isotropic, and the images were reconstructed using zero padding to 0.5 mm isotropic. Cardiac data were acquired by a finger pulse oximeter, and flow data were retrospectively binned into 20 cardiac phases for a temporal resolution of 55 ± 5 ms. Other parameters included an 8° flip angle, a 3 ms echo time, a 5.4 ms repetition time, 4 signal averages, field of view of 220 mm, encoding velocity of 80 mm s^−1^ and acceleration factor of 8. The coverage began just below the C2 segment of the ICAs (roughly at the carotid canal) and ended a few centimetres above the circle of Willis (CoW) for a mean slice coverage of 70 mm, targeting a total scan time of approximately 8 min.

### 2.2. 4D flow processing

**Figure 1.**
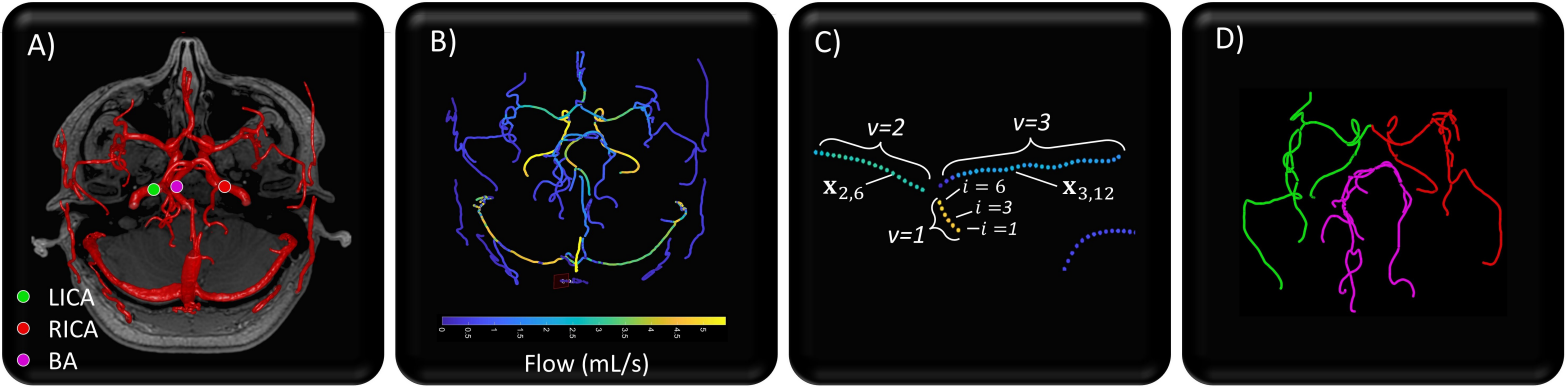
(A) QVT segmentation of cerebrovasculature from 4D flow data identifying root sampling locations for each subvascular tree. (B) QVT visualization of vessel flow. (C) Identification of QVT centreline point of interest by labelled vessels v, and branch locations i. (D) Results of QVT+ to connect vascular trees from initialization of left (green) and right (red) internal carotid arteries (ICA) and the basilar artery (BA) (magenta) initialization points.

The 4D flow data were processed using a modified Quantitative Velocity Tool (QVT+) developed in Matlab v2023a (MathWorks, Natick, MA, USA) (available at https://github.com/ABI-Animus-Laboratory/QVTplus) built on the original QVT [17,18], a semi-automated workflow to process 4D flow data. Baseline QVT processing performs background correction, automatically segments identifiable vasculature, computes vessel centrelines, performs in-plane segmentation and computes cross-sectional flow (for more details, see Roberts *et al*. [18]). QVT+imports DICOM data and contains an updated workflow for the present analysis to compute PWV (see example QVT processing in figure 1).

### 2.3. Measuring pulse wave velocity

For the following mathematical description, we identify a cross-section of interest within the vasculature by its centreline location, $x_{v,i}\in R^{3}$, where v identifies the vessel and i identifies the cross-section within the vessel (see figure 1C for a visual explanation). The set of all centreline points is V and the blood flow field is q where q:V×[1,S]→R, where S is the number of cardiac phases sampled during the cardiac cycle. Thus, q(x,t) is the cross-sectional flow at the spatial location x with dependence on v and i (omitted for the sake of readability), and t∈Z indicates the dependence of flow on the phase of the cardiac cycle.

PWV was measured using three techniques: $c_{wo}$ proposed by Björnfot *et al*. [10], $c_{cc}$ proposed by Fielden *et al*. [19] and $c_{tu}$ described in Redheuil *et al*. [20]. We then ensemble these methods to report PWV vascular territory differences.

#### 2.3.1. Computing distance

All methodologies applied in this work require an estimation of distance to compute PWV. We have previously described the methodology for computing distance from QVT+ data [12]. In brief, the technique requires the selection of a root vessel $x^{r}$ to which all distances will be computed. The corresponding $x^{r}$ vessel is then connected to downstream vessels using a breadth-first search algorithm, which connects nearby vessels, iterating until no new vessels are found from previously connected vessels. The distance for each point $d(x,x^{r})$ was then assigned as the accumulated Euclidean distances from the root vessel $x^{r}$ to each point downstream following vascular connectivity.

For the sake of analysis, we define three cerebrovascular arterial networks stemming from the left and right ICAs and the BA (see figure 1D for visualization of the subvascular trees and corresponding cerebral territory). Although communicants may connect them, we extract a representative PWV from each territory, which has revealed differences in other haemodynamic risk indices [12]. To select $x^{r}$ for each network, the left and right middle cavernous segment (C3) of the ICAs was chosen for the left and right ICAs, and the BA was sampled at the same height as the sampling locations of the ICA (see figure 1A for example locations). To prevent vessel network crossover, manual tagging was performed to exclude communicating vessels and undifferentiable left and right anterior cerebral arteries, as described in the QVT+ workflow on Github and our previous work [12].

#### 2.3.2. Method 1: waveform optimization

To compute $c_{wo}$, the existence of a global waveform (Q(t)) is proposed which can be compared at each spatial location by time shifting the waveform using $d(x,x^{r})$ and the sought PWV. Both Q(t) and $c_{wo}$ are optimized simultaneously by minimizing the least-squares error between the global waveform and all network flow traces as


(2.1) $$\left(c_{wo},Q\right)=\underset{\left({\hat{c}}_{wo},\hat{Q}\right)}{\arg\min}\sum_{x\in V_{x^{r}}}w(x){\left(\bar{q}(x,t)-\hat{Q}\left(t+\frac{d\left(x,x^{r}\right)}{{\hat{c}}_{wo}}\right)\right)}^{2},$$

where $\bar{q}$ is the flow after mean subtracted and normalized by its cardiac cycle s.d. and $V_{x^{r}}$ is the set of all downstream vessel centreline points to $x^{r}$. Linear interpolation is used to predict the time-shifted Q following the original methodology [10]. Lastly, $w\left(x\right)$ is a weight term related to the measurement uncertainty.

#### 2.3.3. Method 2: cross-correlation

To compute $c_{cc}$ for each network, the vessel root ($x^{r}$) flow trace is chosen as a base waveform. The correlation of this waveform $q(x^{r},t)$ is then maximized with each other waveform q(x,t) in the network by optimizing the time-shift ($\Delta t_{cc}$) of the base waveform as


(2.2) $$\Delta t_{cc}(x)=\underset{\left(\Delta {\hat{t}}_{cc}\right)}{\arg\max}\rho \left(q(x,t),q\left(x^{r},t+\Delta {\hat{t}}_{cc}\right)\right),$$

where ρ is the cross-correlation function. To identify temporally precise $\Delta t_{cc}$, q is temporally upsampled to 500 phases at each $x\in V_{x^{r}}$ prior to calculating [4,14,19]. $\Delta t_{cc}$ for all waveforms is then linearly regressed against $d\left(x,x^{r}\right)$ as


(2.3) $$\left(c_{cc},\beta \right)=\underset{\left({\hat{c}}_{cc},\hat{\beta }\right)}{\arg\min}\sum_{x\in V_{x^{r}}}w(x){\left(\frac{1}{{\hat{c}}_{cc}}d\left(x,x^{r}\right)+\hat{\beta }-\Delta t_{cc}(x)\right)}^{2},$$

where β is the time offset to the $x^{r}$ time of zero and yielding $c_{cc}$. Before linear regression, outlier data are removed using a generalized extreme student deviate identification procedure [21]. As with $c_{wo}$, the data can be assigned a weight, $w\left(x\right)$.

#### 2.3.4. Method 3: time-to-upstroke

To compute $c_{tu}$ the same temporally upsampled q is used as in $c_{cc}$ and the time of maximum acceleration defines the upstroke timing:


(2.4) $$t_{tu}(x)=\underset{\left({\hat{t}}_{tu}\right)}{\arg\max}\frac{q\left(x,{\hat{t}}_{tu}+1\right)-q\left(x,{\hat{t}}_{tu}\right)}{\Delta t},$$

which is then used instead of $\Delta t_{cc}$ in a linear fit to distance in equation (2.3) to compute $c_{tu}$.

Challenges in cerebral vessels (unlike the aorta and internal carotids where this technique has been applied) are the significant dampening of flow peak, potential wave dilations or contractions at bifurcations, wave interference from redundant pathways (e.g. communicating arteries), and partial volume effects and low velocity-to-noise ratio in smaller vessels, all altering the waveform. These effects may reduce the spatial and temporal consistency of the calculated maximum acceleration and cause temporal jumps between competing maximum accelerations. As such, we impose some continuity to identify $t_{tu}$. We use the vessel root flow trace ($q(x^{r},t)$) to identify a baseline $t_{tu}\left(x^{r}\right)$, and then, when analysing the downstream waveforms, the closest peak of maximum acceleration to this baseline time is used as the $t_{tu}\left(x\right)$.

### 2.4. Applying different weights

The weights involved in the optimization of the presented PWV techniques (see equations (2.1) and (2.3)) scale the contribution of different waveforms based on their reliability (or quality). In the literature, two weight functions have been proposed to model the data quality. The first was proposed by Björnfot *et al*. [10] as $w_{1}=A(x)\sigma^{2}(x)$, where A is the cross-sectional area and $\sigma^{2}$ is the variance of the flow trace. A second weight function was proposed by Dempsey *et al*. [12] as


(2.5) $$w_{2}(x)=\frac{2}{3}\left(\mu_{\text{circ }}+\frac{\sigma_{q_{\text{mean }}}}{\mu_{q_{\text{mean }}}}+\frac{\sigma_{\text{area }}}{\mu_{\text{area }}}+\frac{\Delta q}{\mu_{q_{\text{mean }}}}+0.5\right),$$

where


(2.6) $$\Delta q=\frac{1}{S}\sum_{t=1}^{S}\left(\max_{-2\;\leq \;j\;\leq \;2}\left(q\left(x_{v,i+j},t\right)\right)-\min_{-2\;\leq \;j\;\leq \;2}\left(q\left(x_{v,i+j},t\right)\right)\right).$$

The four terms in equation (2.6) account for vessel segmentation circularity, flow consistency, area consistency, and waveform consistency over a local five-point stencil where $\mu_{\left(∙\right)}$ and $\sigma_{\left(∙\right)}$ are the mean and standard deviation of the physical quantity (∙). The 0.5 offset and the scaling factor of 2/3 normalize the range between 0 and 1 for acceptable quality data. Any values of $w_{2}§amp;lt;0$ identify extremely low-quality data which are excluded from the fitting procedure. Given their differences, we will evaluate PWVs with both proposed weights in addition to no weight function ($w_{0}$) to see if PWV differences are manifested.

### 2.5. Pulse wave velocity ensemble technique

Finally, it is expected that each method may measure a different PWV [1]. To enhance the assumed reliability of PWV with no clear ideal or validated technique, it is recommended to average the estimations if all are similar, exclude a measure if one is different from the other two, or not have a PWV estimate if all poorly fit or differ [14]. As such, if two or more measurements were within 1.35 m s^−1^, they are averaged; otherwise, we report that the measurement is inconclusive. The threshold 1.35 m s^−1^ is chosen based on the estimation of the propagated PWV error in Markl *et al*. [13], which is also approximately the mean bias of the $c_{cc}$ method compared to the gold standard 2D PC techniques [14]. For statistical purposes, we exclude the estimate if PWV is larger than 50 m s^−1^ or negative (likely non-physiological values) to avoid biasing our measurements. The maximum PWV threshold of 50 m s^−1^ is chosen based on the maximum found in existing literature [4] with most PWVs ranging between 5 and 20 m s^−1^ [5,8–11].

## 3. Results

### 3.1. Comparison of pulse wave velocity techniques and weights

**Figure 2.**
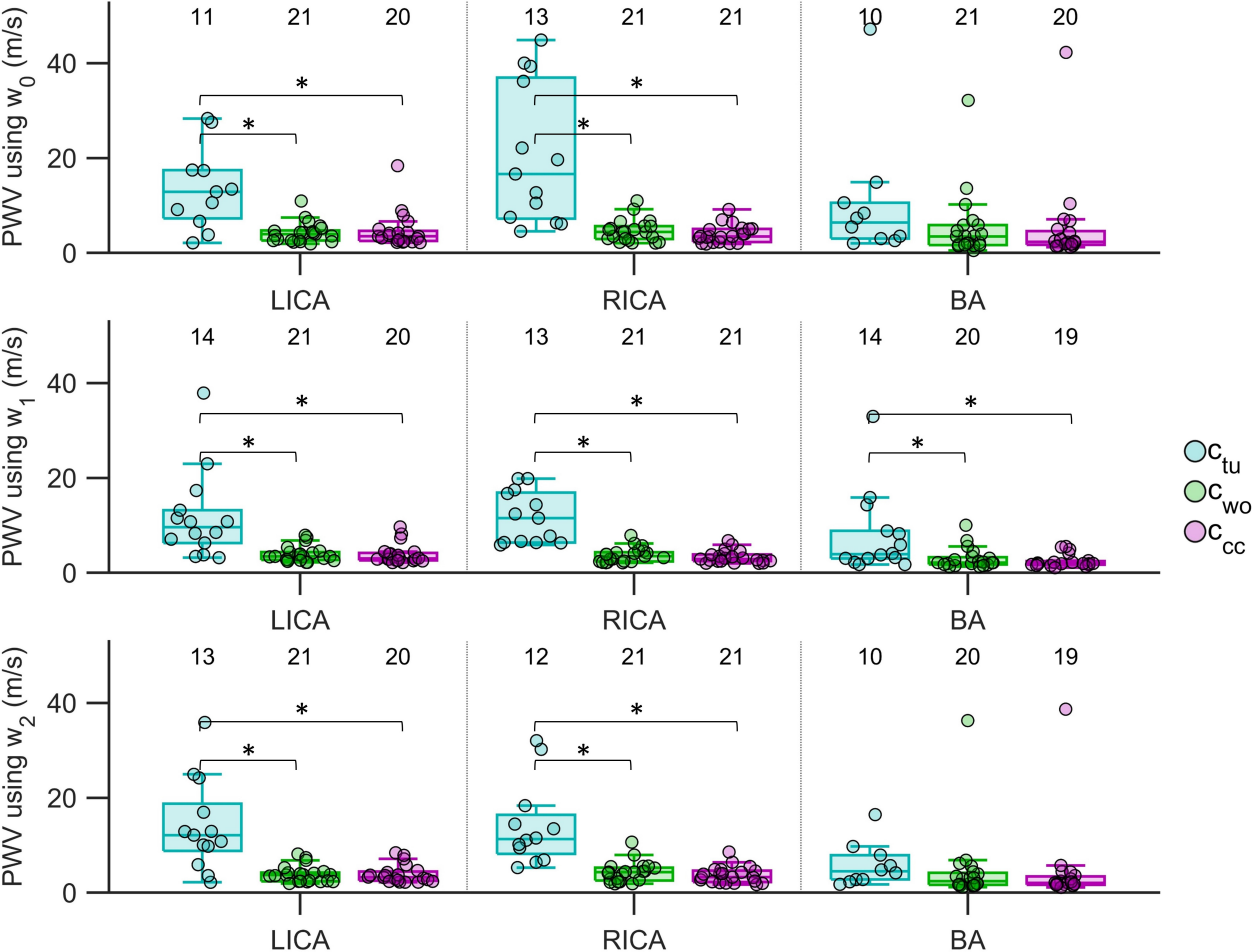
Comparison of PWV techniques for each weight and vascular territory. The numbers above each box plot identify the number of samples with a maximum of 21 for each root (20 in the LICA for $c_{cc}$ and $c_{tu}$). A box with fewer implies that a technique had a failure in measurement. The starred bars identify significant differences between techniques using an *α* < 0.05 significance. Abbreviations: basilar artery (BA), left internal carotid artery (LICA), pulse wave velocity (PWV) and right internal carotid artery (RICA).

On comparing different PWV techniques, we first identified that $c_{tu}$ measured significantly higher PWVs than $c_{wo}$ and $c_{cc}$ in the ICAs using either weight function (see figure 2). This was also observed in the BA using $w_{1}$. $c_{tu}$ also had a significantly higher number of excluded PWVs for any weight: 29/62, 0/63 and 1/62 for $c_{tu}$, $c_{wo}$ and $c_{cc}$ using $w_{0}$, 22/62, 1/63 and 2/62 using $w_{1}$, and 28/63, and the same 1/63, and 2/62 exclusions as $w_{1}$, when using $w_{2}$. One participant’s root ICA landmark, required for measuring $c_{tu}$, and $c_{cc}$, could not be identified. As a result, that vascular territory was omitted from the analysis, leading to a difference in the total number of vessels (62 versus 63) for the different techniques. Concerning the excluded cases, in all instances an exclusion in one method was never predicted in similar magnitude by another method, supporting our choice to exclude these values. All PWV data including excluded cases are tabulated in electronic supplementary material, note 1.

**Figure 3.**
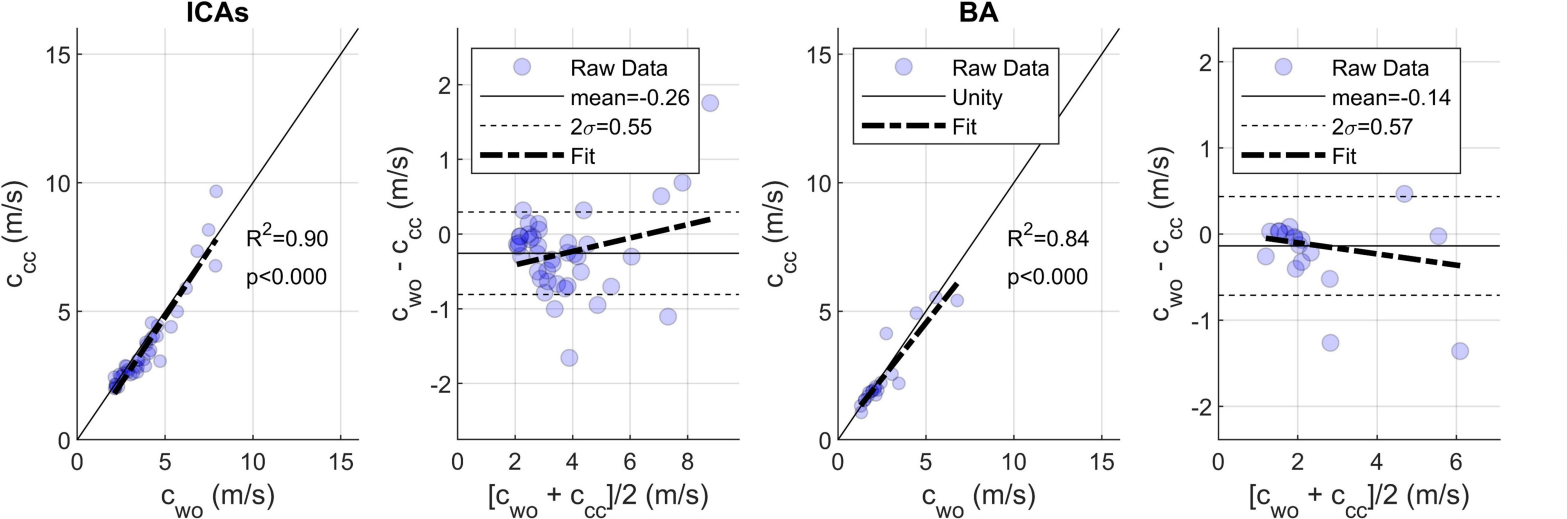
Bland–Altman comparison of $c_{wo}$ and $c_{cc}$ for the internal carotid arteries (ICAs) (left) and basilar artery (BA) (right), both for $w_{1}$.

Focusing on $c_{wo}$ and $c_{cc}$ which displayed similar magnitudes for all roots regardless of weight function (figure 2), we conducted a Bland–Altman analysis to compare the impact of $w_{0}$, $w_{1}$ and $w_{2}$ (see an example comparison figure 3). This analysis (summarized in table 1) showed that $w_{1}$ reported the largest agreement with the smallest bias and standard deviation for the ICAs and BA (although $w_{2}$ performed nearly as well). $w_{0}$, however, had a much lower adjusted *R*^2^ value, and over double the variability versus either $w_{1}$ or $w_{2}$. After applying the 1.35 m s^−1^ exclusion and in a repeated the Bland–Altman analysis, all weights performed similarly in terms of $c_{wo}$ and $c_{cc}$ agreement. Notably, however, the number of PWV values that passed the 1.35 m s^−1^ exclusion varied by weight function. Both $w_{1}$ and $w_{2}$ showed a similar number of agreeing measurements (56/60 and 54/60, respectively), whereas $w_{0}$ had fewer (46/60). Generally, we also observed that the disagreement between techniques increased as the magnitude of PWV increased, independent of weight function.

**Table 1.** Summarized Bland–Altman statistics for and using different weight functions before and after excluding PWVs that differ beyond 1.35 m s^−1^, where *n* identifies the number of measurements included in the calculation.

**u**sing all PWVs					
root	weight	*n*	*R* ^2^	bias (m s^−1^)	2σ (m s^−1^)
ICA	$w_{0}$	41/41	0.75	−0.18	1.49
	$w_{1}$	41/41	0.90	−0.26	0.55
	$w_{2}$	41/41	0.89	−0.31	0.64
BA	$w_{0}$	19/19	0.61	0.61	1.55
	$w_{1}$	19/19	0.84	−0.14	0.57
	$w_{2}$	19/19	0.80	−0.30	0.70

**u**sing PWVs within **1.35 m s**^**−1**^ agreement					
**r**oot	**w**eight	***n***	***R*** ^**2**^	**b**ias (**m s**^**−1**^)	2σ (**m s**^**−1**^)
ICA	$w_{0}$	31/41	0.90	−0.17	0.44
	$w_{1}$	39/41	0.92	−0.27	0.40
	$w_{2}$	37/41	0.93	−0.24	0.43
BA	$w_{0}$	15/19	0.91	−0.24	0.46
	$w_{1}$	17/19	0.90	−0.16	0.36
	$w_{2}$	17/19	0.91	−0.42	0.49

**Figure 4.**
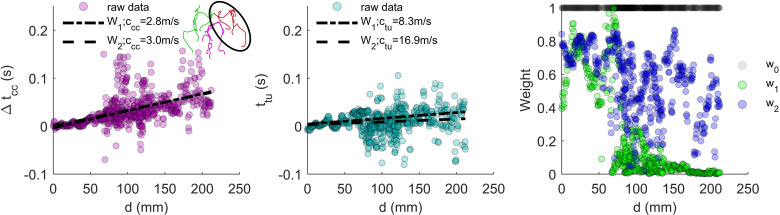
Visualization of wave travelling time delays sorted by distance (d), for the estimation of pulse wave velocity in a nominal vascular territory, in this instance, a left internal carotid artery. For the maximized cross-correlation times, $\Delta t_{cc}$ (left), and time-to-upstroke, $t_{tu}$ (middle), time delays are zeroed with respect to the network root. A comparison of $w_{0}$, $w_{1}$ and $w_{2}$ used for optimization is also shown (right).

To better understand the cause of more frequent $c_{tu}$ exclusion, and the impact of weight functions, we visualize a nominal fit of $c_{cc}$ and $c_{tu}$ for both weights of the left ICA vascular territory in figure 4. By looking at the spatial development (from proximal to distal travelling along the vessel axes), we noticed a higher estimate consistency for $c_{cc}$ than $c_{tu}$ among all data. The source of inconsistency for $c_{tu}$ is likely the greater sensitivity to intrinsic high-frequency contributions which we will discuss later. We also consistently identified a shallower initial slope, which then began to increase approximately 4 cm deep into the vascular network. This corresponds to the shift of carotid canal data to completely intracranial. A similar shallow initial slope was also seen for the BA network, although only in the first few centimetres of data. In all cases, $w_{1}$ consistently attributed most weight for optimization to the proximal vasculature, unlike the weight of $w_{2}$, which is more balanced across the vasculature—while still decreasing—leveraging distal information from the imageable intracranial vasculature (see right panel of figure 4).

### 3.2. Vascular territory pulse wave velocity trends

No significant differences were observed between the left and right ICAs for any weight using a paired *t*‐test which—when combining left and right ICAs—measured a mean PWV of 3.64 ± 1.47 m s^−1^ across all weights (see table 2 for slightly varying values for each individual weight). The BA measured a significantly lower PWV (*p* = 0.01) than the ICAs of 2.53 ± 1.39 m s^−1^ with minor variability between weighting functions. The BA data were clearly more prone to inconclusive measurements when using $w_{1}$ and $w_{2}$, agreeing only in 81% of the cases for BA versus approximately 93% in the ICAs. Inconclusive measurements were roughly even using $w_{0}$. Of the failed BA cases, only two had imageable communicating arteries. Four participants with one or more communicating arteries measured conclusive PWV estimates.

**Table 2.** Summarized *p*-values from correlations of pulse wave velocity with individual covariates of interest for each of the three vascular territories analysed, in addition to considering all data combined. Significant *p*-values are bolded and *n* identifies the number of measurements included in the calculation. Abbreviations: basilar artery (BA), cerebral blood flow (CBF), cross- sectional velocity (CSV), heart rate (HR), left internal carotid artery (LICA) and right internal carotid artery (RICA).

	LICA	RICA	BA	combined
$w_{0}$				
PWV (m s^−1^)	3.59 ± 1.36 (*n* = 16)	3.64 ± 1.48 (*n* = 15)	2.58 ± 1.54 (*n* = 14)	
covariate	*p*-value of trend			
age	0.298	0.128	0.110	0.265
HR	0.158	0.139	0.328	**0.015**
CBF	**0.007**	0.553	0.456	0.155
μ CSV	**0.030**	0.301	0.329	0.074
μ area	0.745	0.206	0.516	0.055
$w_{1}$				
PWV (m s^−1^)	3.72 ± 1.52 (*n* = 19)	3.48 ± 1.43 (*n* = 20)	2.36 ± 1.20 (*n* = 17)	
covariate	*p*-value of trend			
age	0.148	0.641	0.126	0.067
HR	0.274	0.440	0.465	0.132
CBF	0.562	0.769	0.090	0.412
μ CSV	0.126	0.311	0.833	**0.002**
μ area	0.116	0.145	0.675	0.400
$w_{2}$				
PWV (m s^−1^)	3.88 ± 1.84 (*n* = 19)	3.55 ± 1.25 (*n* = 18)	2.66 ± 1.42 (*n* = 17)	
covariate	*p*-value of trend			
age	0.877	0.257	0.707	0.371
HR	0.184	0.083	0.132	**0.01**
CBF	0.672	0.832	0.163	0.463
μ CSV	0.469	0.365	0.875	0.16
μ area	0.675	0.065	0.910	**0.028**

**Figure 5.**
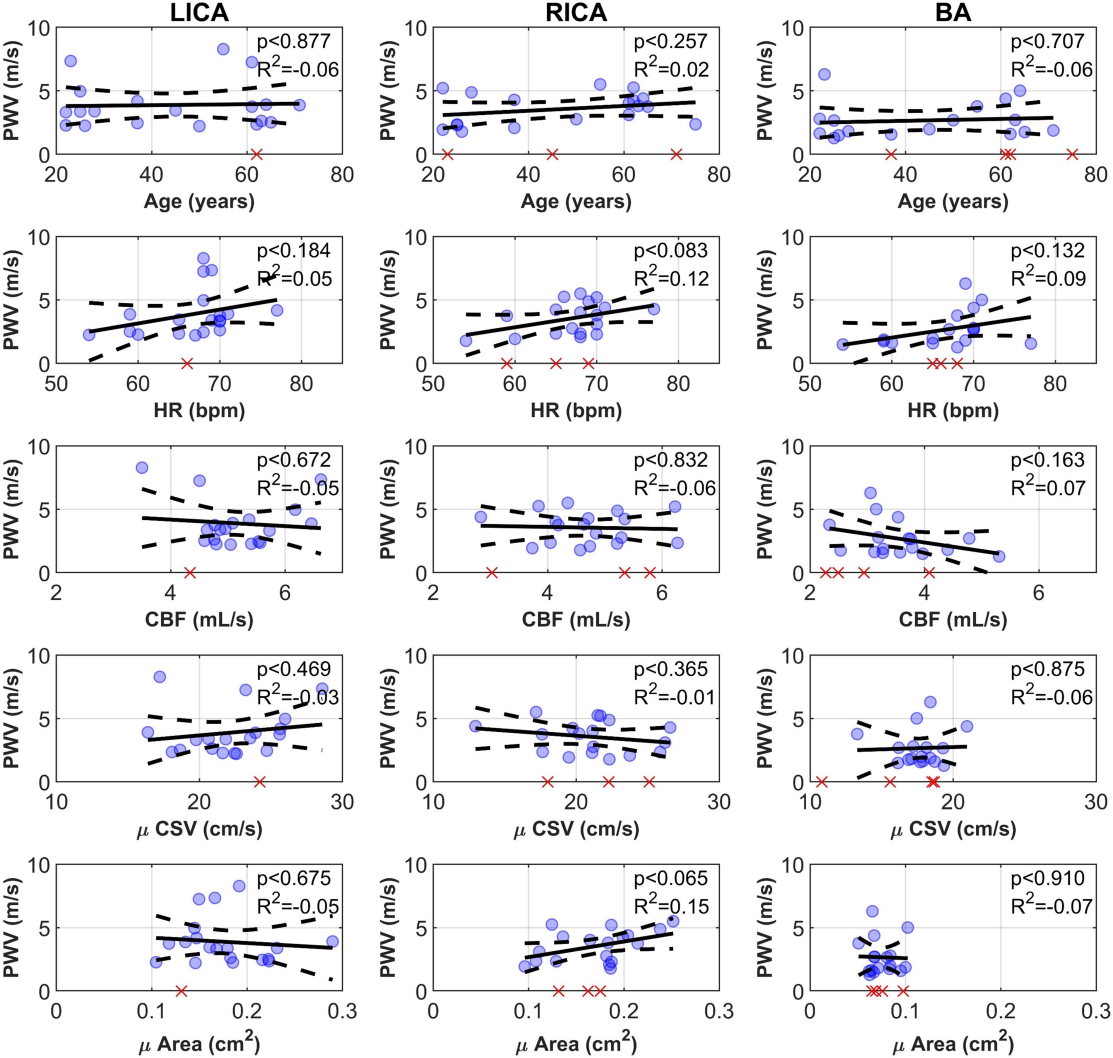
LICA, RICA and BA differences in measured PWV compared against potential covariates: age, HR, CBF, mean CSV and mean CSA. Red crosses are inconclusive PWV estimates. Abbreviations: basilar artery (BA), cerebral blood flow (CBF), cross-sectional area (CSA), cross-sectional velocity (CSV), heart rate (HR), left internal carotid artery (LICA), pulse wave velocity (PWV), and right internal carotid artery (RICA).

Each vascular territory was then correlated against potential covariates for each weight. Covariates included age, HR, as well as more exploratory measures such as CBF, cross-sectional velocity (CSV) and vascular area. Territory CSV and area were approximated by averaging velocity and area over the first 100 mm for each vascular tree. Generally, no vascular territory trended significantly with any covariate except for positive trends with CBF and CSV in the left ICA using $w_{0}$ (see figure 5 for visualization of trends with each territory, and results tabulated in table 2). Data from all PWVs from each territory were then combined and evaluated to enlarge the sample size assuming the vessels are considered haemodynamically distinct. This revealed other weight-specific trends: significant positive correlations were observed with HR using $w_{0}$ and $w_{2}$, CSV was positively correlated using $w_{1}$ and vascular area was positively correlated using $w_{2}$.

## 4. Discussion

This work applied different techniques to measure intracranial PWV to find agreement in a field that has, mainly, measured PWV using different techniques. We identified that two techniques, $c_{wo}$ and $c_{cc}$, agreed remarkably well, while $c_{tu}$ estimated larger PWVs. Differences between possible weighting functions during measurement were minimal after ensembling methodologies. The choice of weight function did not largely influence mean magnitude, but did reveal varying significant covariate trends, highlighting the variability of cerebral PWV.

### 4.1. Enhanced interpretations in literature

Importantly our results consolidate discrepancies in the literature by first identifying $c_{tu}$ magnitude different between other techniques. This applies to the results of Rivera-Rivera *et al*. [4] which reported unprecedented PWVs of 17.9 ± 7.4 m s^−1^ (age 73 ± 7 years, *n* = 42). It was discussed that their cohort was older than that of Peper *et al*. [9] who reported 7.9 ± 2.4 m s^−1^ (age 62 ± 10, *n* = 10), which may explain the increase. However, results from Björnfot *et al*. [10] in a more similar age range also reported consistently lower PWVs of 10.7 m s^−1^ (9.2−13.6 interquartile range) (age range 70–91, *n* = 35). Lastly, Kröner *et al*. [8] reported 6.9 ± 1.5 m s^−1^ (age 56 ± 6, *n* = 16). Rivera-Rivera *et al*. [4] also posited that PWV increases with increasing depth in the brain to explain their larger PWV results, but the work of Björnfot *et al*. [10] and Rivera-Rivera *et al*. [11] used a deeper depth of vasculature and did not report large PWVs, nor did our own work here. A final potential source of disagreement discussed by Rivera *et al*. [4] is that their work used extremely fine temporal sampling, which showed increased PWV compared to standard 20 cardiac phase 4D flow (although their standard 20 phase PWV results were still high). However, Peper *et al*. [9] used a similar fine temporal sequence with 2D PC and did not estimate large PWVs. Based on our results, we can mainly attribute the difference to methodology and note that $c_{tu}$ had not been directly compared with another methodology before now using PC MRI. The authors also used only two points (spatial locations) for their linear regression, while we used multipoint regression in this work, which is expected to improve the precision and consistency of the measurement [1].

The reason for methodology disagreement is likely based on how much waveform information is used to inform the PWV estimation. $c_{tu}$ tracks a specific feature in the slope of the wave while both $c_{wo}$ and $c_{cc}$ are tracking the overall waveform. This makes $c_{tu}$ more susceptible to degradation due to noise and/or interference. Intracranially, waveform interference is expected over the vascular network through communicating arteries and other redundant pathways from the root arteries. This interference may result in the addition of extra modes in the waveform, misleading the tracking of local landmarks. Changes in the waveform due to changes in compliance or cross-sectional area will change the relative position of the upstroke landmark in the overall waveform shape. Low velocity-to-noise ratio may cause spikes in the signal and non-smooth waveforms. These considerations lead us to hypothesize that $c_{wo}$ and $c_{cc}$ are more robust as tracking is performed by evaluating similarity over the complete waveform. Additionally, using the entire waveform benefits noise reduction when some cardiac phases are noisier than others during collection due to motion or HR variability.

The studies from Björnfot *et al*. [5,10] and Rivera-Rivera *et al*. [11] (all used $c_{wo}$) are, to the best of our knowledge, the only works that include significant intracranial vessel information when reporting PWV. We report significantly lower, and age-independent intracranial PWV results compared to those studies. To explain why, since we have established that $c_{cc}$ and $c_{wo}$ similarly track the global alignment of the waveform and are nearly identical, we used the visualization provided by figure 4 for all cases and identified three important findings: (i) the initial slope of $c_{cc}$ is almost always shallower than intracranially (larger PWV), (ii) $w_{1}$ of Björnfot *et al*. [5,10] assigns almost all optimization weight to the early data, and (iii) 4D flow coverage in Björnfot *et al*. [5,10] and Rivera-Rivera *et al*. [11] was larger inferiorly, covering more of the carotid length and canal. Together, we reason that these study results have a bias towards extracranial PWVs, presenting lower intracranial PWV contributions. This is supported by observations that arterial distensibility in the initial ICA segments is significantly decreased compared to the intracranial segments (in line with our observed slope changes and $w_{1}$ bias) [22]. There is also good agreement of Björnfot *et al*. [5,10] and Rivera-Rivera *et al*. [11] with PWV estimates from Peper *et al*. [9] that also used the $c_{cc}$ technique extracranially. Björnfot *et al*. [5,10] and Rivera-Rivera *et al*. [11] estimated slightly larger PWVs than Peper *et al*., which is likely caused by the methodological averaging of $c_{cc}$ and $c_{tf}$ (not measured here) done by Peper *et al*. [9]; $c_{tf}$ was shown to estimate a smaller PWV than $c_{cc}$ by Markl *et al*. [13], thus lowering their average. Additionally, to support our hypothesis of extracranial bias in those works, we imaged an additional subject with a large extracranial coverage and observed supporting results showing extracranial increase in PWV (see electronic supplementary material, note 2).

Visualization of $w_{1}$ also helps to explain the results in Björnfot *et al*. [5]. There, the authors showed how PWV changed as the slice coverage from the CoW was gradually extended inferiorly and superiorly. At first, the PWV was quite variable with low coverage, and as coverage was extended, the PWV decreased (closer to our values). As coverage expanded further, PWV began to rise. Our interpretation is that in the beginning the variability is explained by the low cross sections and variable time delays shown in figure 4. As coverage increases, the measurement becomes more accurate for intracranial PWV and the mean PWV decreases, but eventually, as coverage includes more carotid canal and extracranial data, the weight to intracranial data decreases and the PWV increases. This highlights the problem with $w_{1}$. When the authors proposed it, there was no explanation or validation. It makes sense to weigh larger areas that have more voxels and better flow approximations, but the rationale behind the variance factor, $\sigma^{2}$, in $w_{1}$ is unclear. As blood flows through the cerebrovasculature, the flow becomes more steady, but that does not mean that the data are less useful or reliable as $w_{1}$ suggests. $w_{1}$ also does not factor in poor luminar segmentations or locally noisy data like $w_{2}$ does by considering local stability or excluding inconsistent cross-sectional area changes, which is a natural consequence of automated processing techniques [12]. However, since Björnfot *et al*. [10] used a different 4D flow processing platform and sequence, we are not saying they encountered data that should have been excluded when performing this type of analysis, just that $w_{1}$ generates a significant bias when analysing intracranial vasculature.

Finally, we observed a similar deviation between PWV methods as a function of magnitude as Peper *et al*. [9], and, as such, emphasize that care should be taken when interpreting PWV results greater than 7.5 m s^−1^.

### 4.2. Interpreting vascular territory and covariate trends

#### 4.2.1. Age

**Figure 6.**
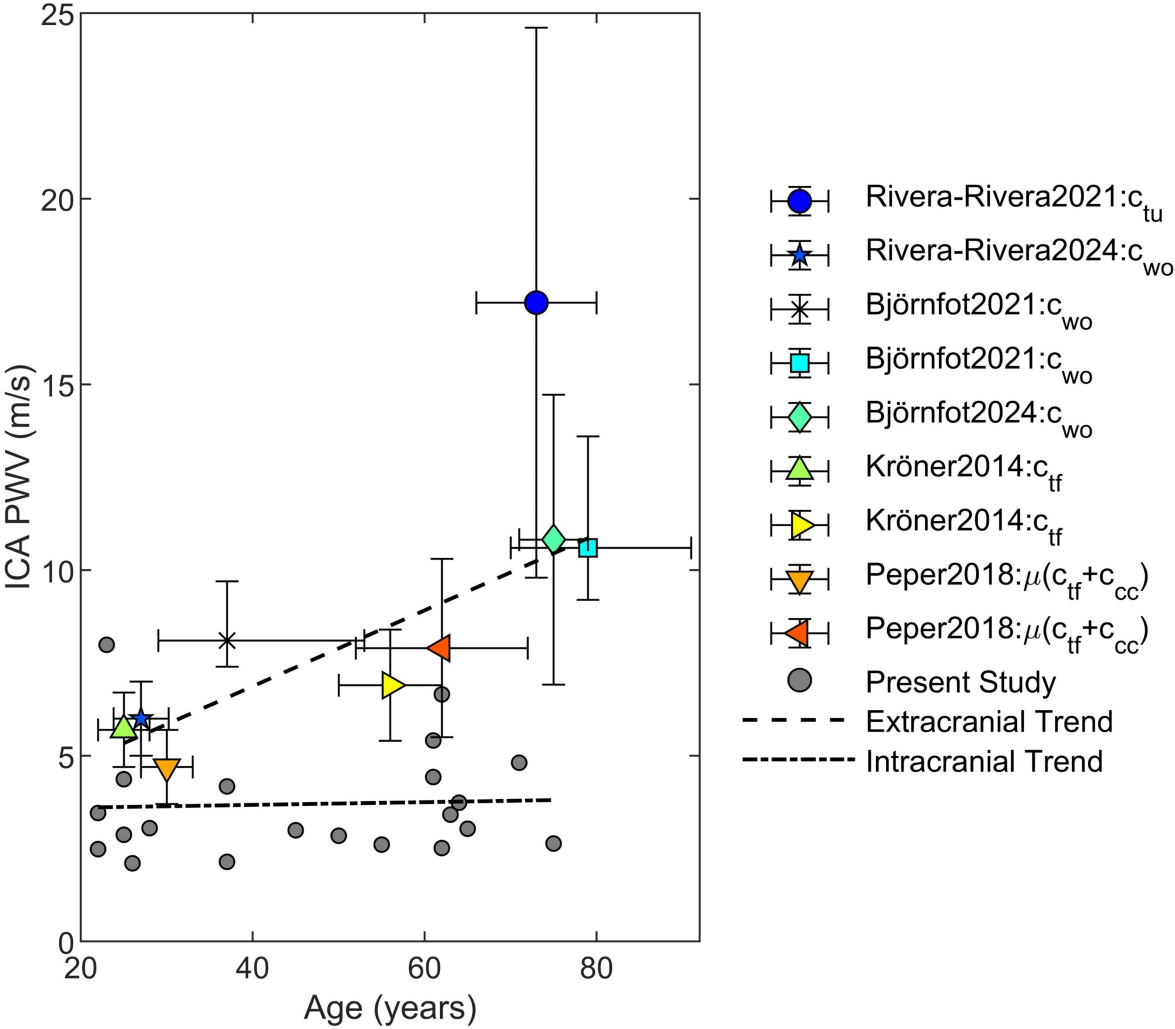
Comparison of the present mean internal carotid artery results using $w_{2}$ versus literature. Deviation is either s.d. or interquartile range depending on how the results were presented in the original works. Linear fit trends used a least squares optimization scheme using the inverse of the mean deviations as weights for the literature trend, and equal weight for this study.

Unlike any internal carotid vasculature work to date, we did not observe a dependence on age with PWV using any weight which has previously been clearly demonstrated with similar sample sizes using the same techniques used here [9,10] (see figure 6 and table 3 for summarized literature findings). The main anticipated difference is coverage and weight function, with other work proposed in the previous section as extracranially biased, whereas here there is an intracranial focus. Two possible explanations are: (i) intracranial vessels do not age similarly to other systemic vessels where clear age trends are measured [2] or (ii) there are other anatomical or physiological influences that alter PWV intracranially. We support the latter explanation and hypothesize that extravascular stiffness influences the perceived compliance of intracranial vessels that PWV is hypothesized to reflect. Compared to other tissue types the brain is extremely soft [23], due in part to its large fat content. This contrasts with the extracranial carotid vasculature that is surrounded by stiffer muscle tissue, fascia, or even bone through the carotid canal. These extravascular mechanics may contribute to the measured PWV. In addition, the intracranial vasculature is also modulated by neurons and glia in complement to autoregulatory and autonomic influences that can alter vascular tone [6]. These factors may play a role in preserving low PWV during healthy ageing. From a physiological perspective, this age independence is a positive mechanism that would prevent pulse wave encephalopathy in deeper brain tissue by allowing more time to dampen the wave [24,25]. It is possible that older individuals in the cohort were unusually healthy. However, this same cohort’s mean CBF, pulsatility index, damping, and transmission have previously been reported on, all of which showed significant dependences with age [12], supporting this cohort’s suitability for drawing conclusions. Also of interest is that $w_{1}$, which we hypothesize is extracranially biased, trended very close to statistical significance in its association with age. This further suggests that bias towards extracranial vasculature contributes to the observed age dependence.

**Table 3.** Results of previous literature on pulse wave velocity in or near the cranium. [ ] = range, ^†^ = median and *n* identifies the number of measurements included in the calculation.

reference	anatomical location	*n*	sequence	technique	age (years)	mean PWV (m s^−1^)
Peper *et al*. [9]	common carotid to C1 of ICA	23	2D PC	two-point mean of $c_{cc}$ and $c_{tf}$	30 ± 3	4.7 ± 1.0
		10			62 ± 10	7.9 ± 2.4
Kröner *et al*. [8]		16		two-point $c_{tf}$	25 ± 3	5.7 ± 1.0
		16			56 ± 6	6.9 ± 1.5
Björnfot *et al*. [5]	C1 of left and right ICA + downstream vessels	190	4D flow	multipoint $c_{wo}$ using $w_{1}$	79 ± 44 [66–85]	^†^10.82 (3.94)
Björnfot *et al*. [10]		10			37 [29–53]	^†^8.1 (2.3)
		35			79 [70–91]	^†^10.7 (4.4)
Rivera-Rivera *et al*. [11]		10			27 ± 3.2 [21–32]	6.0 ± 0.4 (estimated)
Rivera-Rivera *et al*. [4]	C1–C3 of the ICAs	32		two-point $c_{tu}$	58 ± 5	10 ± 4.8
		42			73 ± 7	17.2 ± 7.4
present study	C2 of ICA + downstream vessels	20		ensembled multipoint ($c_{cc},\;c_{tu}$, $c_{wo}$)	48 ± 18	3.64 ± 1.47

#### 4.2.2. Heart rate

Concerning the positive trend with HR, while no individual vascular territories measured significant trends, PWV increased with HR in all territories and significantly for $w_{0}$ and $w_{2}$ when ICA and BA data were combined. This HR trend is expected: the positive dependence on HR can reflect an increase in sympathetic activity [26], which can also cause an increase in vascular tone [6], making the vessels ‘stiffer’ and accelerating the pulse wave. In an MRI setting, which can be stressful for some participants, this effect can be expected. It may also alter the viscous properties of the vessel, increasing stiffness [27] and has been reported for cerebral vessels in other work [11].

#### 4.2.3. Cerebral blood flow and cross-sectional velocity

Disregarding the significant positive CBF PWV trend observed with $w_{0}$ in the LICA (which is uninterpretable), no significant trends with CBF were observed in this study. As another exploratory covariate, mean CSV trended significantly with $w_{1}$ (again disregarding the LICA $w_{0}$ trend). An increase in PWV results in a wider pressure wave, with a more sustained blood flow across the cardiac cycle but does not necessarily imply an increase in CBF as that also depends on the wave amplitude. Since these techniques do not provide a description of the pulse wave, only an approximation of its velocity, deeper interpretation to the relationship and the weight dependence is left to future work to probe the waveforms themselves.

#### 4.2.4. Area

The dependence on area is also expected based on the theoretical equation for vascular PWV [28]:


(4.1) $$\begin{array}{c} PWV=\sqrt{\frac{A}{\rho }\frac{\partial P}{\partial A}}, \end{array}$$

depending on area (A), pressure (P), and density (ρ). Interestingly, only $w_{2}$ revealed this trend with statistical significance, suggesting that $w_{2}$ is well suited for cerebrovascular data. While $w_{0}$ nearly captured this trend (*p* = 0.055), its natural weighting of distal data included lower-quality measurements (data that $w_{2}$ more effectively excluded) likely obscuring the area dependence.

#### 4.2.5. Internal carotid versus basilar vascular tree differences

It was possible that, due to differences in length from the heart or changes in geometry, the left and right ICAs would have different PWV, but our data suggest they are the same—a reasonable outcome considering their analogous vascular function territory symmetry, and similar haemodynamic symmetry observed in other work [22,29]. We also combined both network data before fitting PWV (not shown) and found that the resultant ICA PWV was also indistinguishable from left and right, so the assumptions of mixing networks in Björnfot *et al*. [5,10] and Rivera-Rivera *et al*. [11] remain acceptable. The BA, however, identified lower PWV than either ICA. The main cause of lower BA PWV is attributed to the dependence on vascular area we identified, but could also reflect differences in mechanical properties or tone. The lower BA PWV supports observations that ICA PWV was not correlated with occipital white matter hyperintensities [5] (assuming elevated PWV is a risk factor for neurodegeneration). However, results from Björnfot *et al*. [5] were generated using $w_{1}$ and only for $c_{wo}$, not averaged by the ensemble technique, so we do not place too much emphasis on these results. However, if correct, our previous work identified significant pulsatility-based risk in the BA, suggesting that pulsatility-dependent neurodegeneration may still impact the region [12]. Significantly larger BA distensibility has also been identified in other work [30], which suggests more compliant vessels, in line with the lower BA PWV measured here.

It is possible that the lower BA PWV is caused by methodological issues. Estimation failed more frequently than in the ICAs, and inconclusive measurements were more common. On visual inspection of inconclusive measurements fits (e.g. figure 4), they were not clearly noisier than conclusive measurements. Coupled with the lack of dependence on communicating arteries leads us to believe that inconclusive measurements are not caused by interference of waves through the posterior communicating arteries, but rather reflect special cases where each technique demonstrates a unique bias. These further highlight that research into an optimal technique is warranted.

### 4.3. Comparing pulse wave velocity with pulsatility-based indices

Recently, these data were processed to analyse pulsatility-based indices [12], and since both pulsatility indices and PWV are proposed to enhance neurovascular haemodynamic evaluation, it is relevant to compare the two. Previously reported were that pulsatility-based indices significantly positively correlated with age and CBF, and negatively correlated with HR. Whereas here (using the same $w_{2}$ as [12]) PWV was positively correlated with HR and vascular area when analysing all territories. We directly compared PWV with vascular pulsatility, the pulsatility damping factor and pulsatility transmission and identified no significant correlations (see plot in electronic supplementary material, note 3). The different covariates and lack of direct PWV versus pulsatility correlation suggest that these indices could be complementary during haemodynamic evaluation and measure different haemodynamics.

### 4.4. Limitations

Our study was carried out in a group of 21 volunteers, and a larger sample size would be required to achieve a higher statistical significance in the population trends presented here to support our claims. Nonetheless, all results obtained by comparing the different methodologies remain valid, and the analysis presented in the previous sections provides a rational basis for understanding the dissimilarities between the PWV techniques.

#### 4.4.1. Method limitations

A general limitation of PWV analysis in cerebrovasculature stems from the application of techniques developed for the aorta. Intracranially, the waveform changes due to interference of multiple waves from different networks, changing the number of modes and amplitude. Simultaneously, decreasing/increasing arterial radius can cause wave contraction/expansion. Although the former is more common downstream, the CoW provides redundant pathways in which both effects may occur. Since the waveform may be subject to these changes, techniques that presume the existence and stability of a global signal (global waveform, consistent maximum acceleration and correlation) may all be failing, masking important features and trends, and may explain the manifestation of inconclusive measurements. Instead, techniques should be developed that track the waveform(s) while accounting for interference, energy dissipation and heterogeneities in vascular compliance across the vasculature. These changes in waveform may explain the variability of time delays we observed, which did appear unbiased, and perhaps current methods are still applicable, but a more comprehensive model for wave tracking should be used to confirm the results. We also only tested the PWV techniques that have previously been used intracranially, and methods such as $c_{tf}$ or time-to-peak are still to be tested. Previous studies have shown that $c_{cc}$ and $c_{tf}$ (but not time-to-peak) perform similarly and agree with 2D PC techniques [13,14]. For this reason, we only used $c_{cc}$ as an effective representation of $c_{tf}$.

Lastly, these methodologies were applied to estimate a single PWV per vascular territory, which may mask pathology in single vessels or regions. This would manifest as nonlinearities in the time-delay plots, such as those observed within the carotid canal in these data. While these techniques could be applied to measure vessel-specific PWV, it remains unquantified how reduced cross-sectional areas might impact precision. Without a ground truth, addressing this is beyond the scope of the current work. If a vessel-specific PWV is sought, an alternative methodology, such as the 2D PC flow-area (QA) method, could be applied [31,32]. This was not tested here as the coarser in-plane resolution of 4D flow limits the accuracy of area dilation even in the largest vessels compared to 2D PC methods [33]. Additionally, this technique assumes unidirectional and reflection-free waveforms which are not anticipated intracranially [28] so while validations of the QA method in phantoms exist [31], they may not directly translate to brain applications and require validation for intracranial use.

#### 4.4.2. Sequence limitations

To the best of our knowledge, this is the first study reporting PWVs in the brain using the kat-ARC sequence. As such, it is entirely possible that the sequence and parameters chosen contribute to the differences between other studies. We do not anticipate that the population trends measured are specific to this sequence. Previous work using this sequence [12] identified pulsatility-based trends with sex, age and CBF that align with findings from other studies using the more well-established three-dimensional radially undersampled (PC-VIPR) sequence [29,34]. The PWV magnitudes, however, may not be comparable between studies; preliminary results of the kat-ARC acceleration factor influence on 4D flow-derived metrics are available in electronic supplementary material, note 4. Complementary results for other scanners and sequences are needed to identify the comparative magnitude difference, however. Future work is warranted to quantify the influence of key sequence parameters (acceleration, spatiotemporal resolution, etc.) and to identify a robust sequence that minimizes acquisition time and enhances clinical adoption.

We acknowledge that using the single, high velocity encoding value leads to lower SNR at low velocities, which may increase time-delay variability in low-velocity vasculature such as the BA and contribute to PWV differences. This was not an observed issue when analysing our time-delay plots; however, testing these methodologies using a dual VENC sequence could help determine whether this influenced the data. Finally, a constant 20 cardiac phases were collected regardless of HR resulting in varying temporal resolutions. Thus, key peak dynamics during systole may have been missed in some participants. Combined with the generally long acquisition times, this results in unequal temporal averaging, potentially masking waveform features that contribute to variability in time delays, methodological consistency, and PWV accuracy. Since $c_{cc}$ and $c_{wo}$ rely on global waveform alignment, they may be robust to temporal limitations. It would be interesting to investigate whether the method consistency improves in a cohort with identical and fine temporal resolutions.

### 4.5. Perspective

Our methodology presents an ensemble approach (consensus of 3+ techniques) to measure robust PWV measurements across three main cerebrovascular networks using current techniques. Based on our analysis and the hypothesized differences between intracranial and extracranial dynamics, a logical next step would be to separate data that contain mixed extracranial and intracranial information and to evaluate the differences between these regions. Unfortunately, in our cohort, the data were almost completely intracranial, with coverage beginning approximately above the carotid canal. Importantly, the lack of consistency with thoracic PWV trends with age suggests that intracranial PWV may not be as reflective of arterial stiffness as once thought, and new research to characterize major influencing sources warrants exploration. Validation of these techniques using a realistic phantom would help clarify whether PWV reflects arterial stiffness, or a composite metric influenced by other factors. Complementary approaches, such as magnetic resonance elastography and possibly intracranial pressure measurements, could help explore the role of extravascular stiffness and pressure on PWV. Additionally, measuring PWV with a vasoactive stimulus would help determine the role of perivascular innervation and vascular tone effects. As an index, PWV likely still holds value because it informs on the speed of the pressure wave which dictates the time available for pressure wave dampening before it reaches the microvascular bed—where damage is hypothesized to occur [24,25].

To support analysis by other groups with ideal data, our tools are publicly available on an open-source platform. In this continued work, we recommend ensemble techniques for PWV analysis and the continued use of the 1.35 m s^−1^ threshold. Performing visual inspection of the resulting fit, when possible (e.g. $c_{cc}$), with a particular focus on large PWV values is also recommended. New techniques for measuring PWV that better accommodate dynamic intracranial waveforms deserve exploration. We look forward to the evaluation of intracranial PWV in other cohorts.

## Data Availability

Code is archived in Zenodo (https://doi.org/10.5281/zenodo.14715242) [35]. Concerning scan data, in line with the approved ethics documentation from this study, endorsed by the New Zealand Health and Disability Ethics Committees (NZ HDEC), and in accordance with our indigenous and community engagement policies, non-identifiable data are available upon request and subsequent approval by the Mātai Ngā Māngai Māori Board (contacted an nmm@matai.org.nz). This protocol ensures adherence to ethical standards, respects community involvement, and upholds data sovereignty principles. Supplementary material is available online [36].
